# Attractive curves: the role of deformations in adhesion and friction on graphene†

**DOI:** 10.1039/d2na00283c

**Published:** 2022-09-14

**Authors:** P. V. Antonov, P. Restuccia, M. C. Righi, J. W. M. Frenken

**Affiliations:** Advanced Research Center for Nanolithography Science Park 106 1098 XG Amsterdam Netherlands frenken@arcnl.nl; Department of Physics and Astronomy, University of Bologna Viale Berti Pichat 6/2 40127 Bologna Italy clelia.righi@unibo.it; Institute of Physics, University of Amsterdam Science Park 904 1098 XH Amsterdam Netherlands

## Abstract

Friction force microscopy measurements reveal a dramatic difference of a factor 3 between the friction forces experienced on single-monolayer graphene on top of oxidized and unoxidized copper substrates. We associate this difference with the strong and weak adhesion that the graphene experiences on these two substrates, respectively, but argue that it is too large to be ascribed either to a difference in contact area or to a difference in contact commensurability or even to a combination of these two effects. We use density functional theory to show a significant increase in the chemical reactivity of graphene when it is curved.

## Abstract 2

The increase of chemical reactivity in curved graphene explains the reduction in friction for monolayer graphene over oxidized/unoxidized copper substrates.

## Graphical abstract

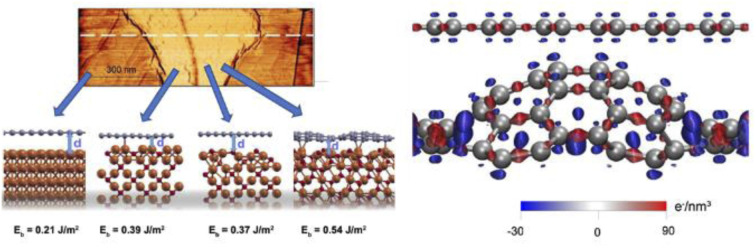

## 1. Introduction

Graphene is a single monolayer of sp^2^ bonded carbon. It is the lightest, thinnest and strongest material known to date^1^ and new, extreme properties continue to be added to the growing list for this special, two-dimensional material. As graphene forms a natural constituent of graphite and graphite is commonly used as a solid lubricant, the frictional properties of graphene have attracted attention too and even a single layer of graphene is known to reduce dry friction and wear in sliding contact.^2–11^ Nanoscale friction experiments with friction force microscopy (FFM) have demonstrated that friction on graphene can depend sensitively on the number of graphene layers^12–14^ and approximately reaches the value of bulk graphite only at a thickness of four layers.^15^

Since graphene has a relatively low out-of-plane bending stiffness, it can easily bulge (pucker) in front of the sliding AFM tip.^15^ This effect was observed specifically on silicon oxide and copper substrates, both of which have weak adhesion to graphene. The puckering was found to be sensitive to the number of graphene layers and to become less pronounced, as the number of layers increases. The latter effect is associated with the stronger adhesion of the graphene top layer to underlying graphene layers, which reduces the puckering and is accompanied by a reduction in the friction force. Correspondingly, the puckering is suppressed on substrates to which graphene experiences strong adhesion, such as mica,^15^ on which the friction force is observed not to depend on the number of graphene layers.

The increase of friction associated with the puckering effect was originally attributed to the increase in contact area between the AFM tip and the fold that it introduces in the graphene in front of itself.^15–17^ Later, the same authors showed that the friction increase was substantially larger than the change in contact area that they estimated due to the puckering of the graphene. Therefore, they proposed an alternative explanation, in which a key role was given to the freedom of the graphene to locally deform in order to optimize its commensurability with those regions on the tip, to which it made intimate contact. This contact-commensurability effect could indeed be recognized in classical MD simulations.^18^ Recent friction force microscopy experiments on free-standing graphene provided further evidence for this scenario.^19^

Recent density functional theory (DFT) calculations revealed that the adhesion of a single layer of graphene to a metallic substrate is highly sensitive to the electronic structure of that substrate. For example, graphene binds more strongly on iron than on copper because the d states of iron, which are partially occupied, rehybridize with π orbitals of graphene, thereby promoting chemisorption instead of physisorption.^20^ These differences have a direct impact on the tribological behavior of graphene on the two different substrates.^21,22^

In this article, we present a combined experimental and theoretical study of the interaction between a graphene monolayer and its support and the influence of this interaction on friction. As supports for the graphene, we used clean copper and oxidized copper, and we find that the friction forces experienced on the graphene on these supports differ by a factor 3 – by far the largest friction contrast found on graphene to date. This difference exceeds the combined effect of the puckering-induced increase in contact area and the deformation-assisted improvement of contact commensurability, mentioned above, and suggests the contribution of an even stronger source of influence on the friction force. We applied density functional theory (DFT) calculations to reveal that the puckering induces a re-hybridization of the carbon bonds within bent graphene regions, which transforms them from inert to ‘reactive’. A tip sliding on such a region therefore experiences higher adhesion and friction.

Our study may be relevant in the context of potential applications of single-layer graphene in dry sliding contacts, especially in cases where local fine tuning of friction forces may be required, *e.g.*, in MEMS or NEMS devices.^23,24^

## 2. Results and discussion

### 2.1. Experimental results

**Fig. 1:**
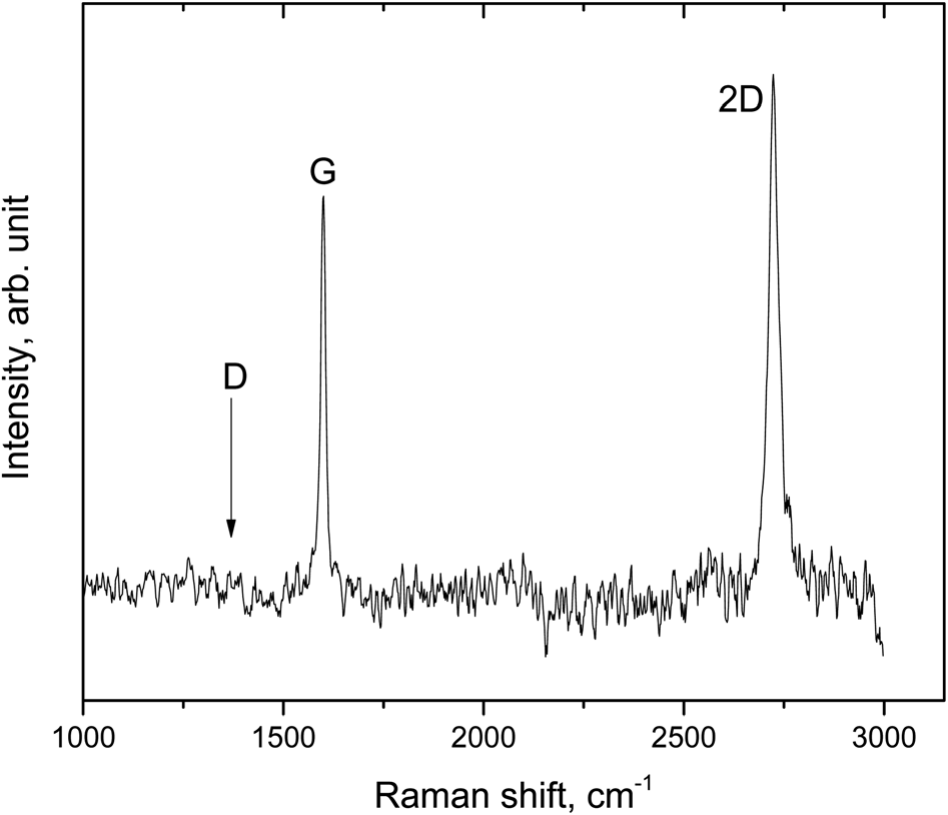
Raman spectrum of an as-grown graphene sample, 10 days after CVD growth of the graphene on a (111)-textured copper film on the (111) surface of sapphire. The G- and 2D-peaks are labeled, as is the location where the D-peak, due to structural defects, should be expected. The spectrum indicates that this is a single graphene layer of relatively high structural quality, *i.e.*, low defect density.

We start by characterizing the freshly deposited graphene-on-copper system, prepared as described in Section 3. Fig. 1 presents a typical Raman spectrum, obtained 10 days after the graphene had been deposited. The symmetric and sharp 2D-peak at 2727 cm^−1^ confirms that this is a single graphene layer, while the absence of a D-peak at 1350 cm^−1^ demonstrates the absence of crystal defects within the graphene grains and should be taken as the signature of the good quality of the material.^25,26^ Also the intensity ratio between the 2D–G-peaks, *I*_2D_/*I*_G_, which is slightly larger than unity, indicates the presence of only monolayer graphene. Note, that this ratio strongly depends on the laser excitation energy and that it is sensitive to the details of the binding between the graphene layer and the substrate.^27^ It was demonstrated that in the green laser range (excitation energy of 2.2 ÷ 2.4 eV) the intensity of the Raman resonance profile becomes stronger than at higher energies (above 2.8 eV). This effect is related to the photoluminescence band of the Cu substrate. In our experiments, we employed a laser wavelength of 514 nm, which corresponds to an excitation energy of 2.41 eV. The acquired spectrum is in good agreement with the literature.^25–27^ For completeness, we mention that the background of the luminescence from the copper substrate was subtracted from the spectrum in Fig. 1.

**Fig. 2:**
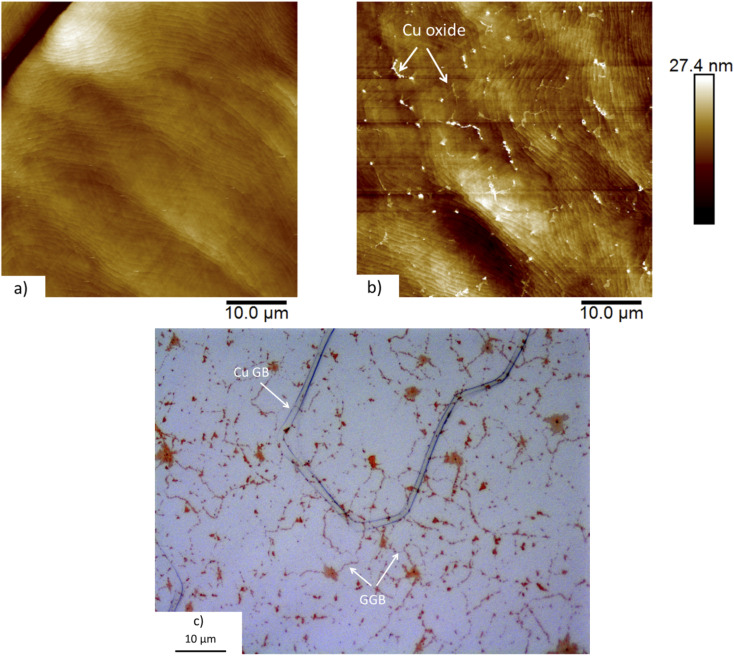
AFM images and optical micrograph of a graphene-covered copper surface. (a) AFM image a few days after graphene deposition and subsequent exposure to air. (b) AFM image of nearly the same surface region after 2 months of additional exposure to air. Note the network of protruding lines and islands, associated with the oxidation of the underlying copper substrate *via* the GGBs. (c) Optical micrograph of a different area of the same surface after 2 months of exposure to air, also showing the network of oxide-decorated GGBs. The image also shows a Cu GB. Note, that the orientations of the GGBs do not seem to be affected by the orientation of the Cu GB.

In addition to the Raman spectra, we inspected the graphene-on-copper samples at length with AFM, as illustrated by Fig. 2a. Defects where the graphene was locally missing have been found on occasion. At these locations, it was possible to measure the height of the overlayer and also those measurements invariably verified the single-monolayer character of our material. Optical inspection and AFM images indicate that the graphene monolayer covered the entire substrate uniformly and that the substrate (plus graphene) was relatively flat (±10 nm).

After deposition and initial characterization, the graphene-on-copper samples were stored at room temperature in air at a relative humidity of approximately 50%, for a total duration of two months. We found that not only bare copper surfaces changed under the influence of the atmosphere, but also the surface topography of our graphene-on-copper samples slowly evolved. This can be recognized by comparing the AFM image in Fig. 2b, taken on the graphene-covered sample after two months of air exposure, with the ‘fresh’ one in Fig. 2a and c shows an optical micrograph of the same, two-month-air-exposed, graphene-covered sample. As mentioned above, the AFM image in Fig. 2a is characteristic for high-quality graphene on relatively flat and smooth copper. It shows a modest height variation and individual, atomic steps of the copper substrate can be distinguished. In addition to these features, Fig. 2b, which shows nearly the same area, reveals a fine network of protruding lines and islands. Typically, the heights of these protrusions do not exceed 5 nm. The optical image of Fig. 2c shows a similar network, of lines and islands where the optical contrast has changed. We associate these slow changes with oxidation of the copper substrate, as will be further substantiated by the AFM and Raman spectroscopy measurements, discussed below. Structurally perfect graphene is impenetrable for oxygen and structural defects in the graphene, such as the graphene grain boundaries (GGBs), form the only locations where oxygen can pass and reach the copper.^28^ This gives rise to a slow, diffusion-limited oxidation of the copper substrate, that sets in at the GGB and spreads out sideways to oxidize the copper underneath the graphene.

For completeness, we mention that the optical image also contains an extended defect that is clearly different in shape and contrast from the oxide-decorated GGBs. We associate it with the copper film and interpret it as a copper grain boundary (GB). The orientations of the GGBs seem not to depend on the orientation of the copper steps and the copper GB (Fig. 2c). The black dots, visible mostly on the copper GB in the optical micrograph (Fig. 2c), are regions of bulk copper oxide that have grown in height above the sample surface and are not dressed with graphene anymore. We conclude this from Raman measurements conducted at these locations. Presumably, these regions are formed at places where the highest defect density, both in the graphene and in the underlying copper, enables a relatively high oxidation rate.^28^

**Fig. 3:**
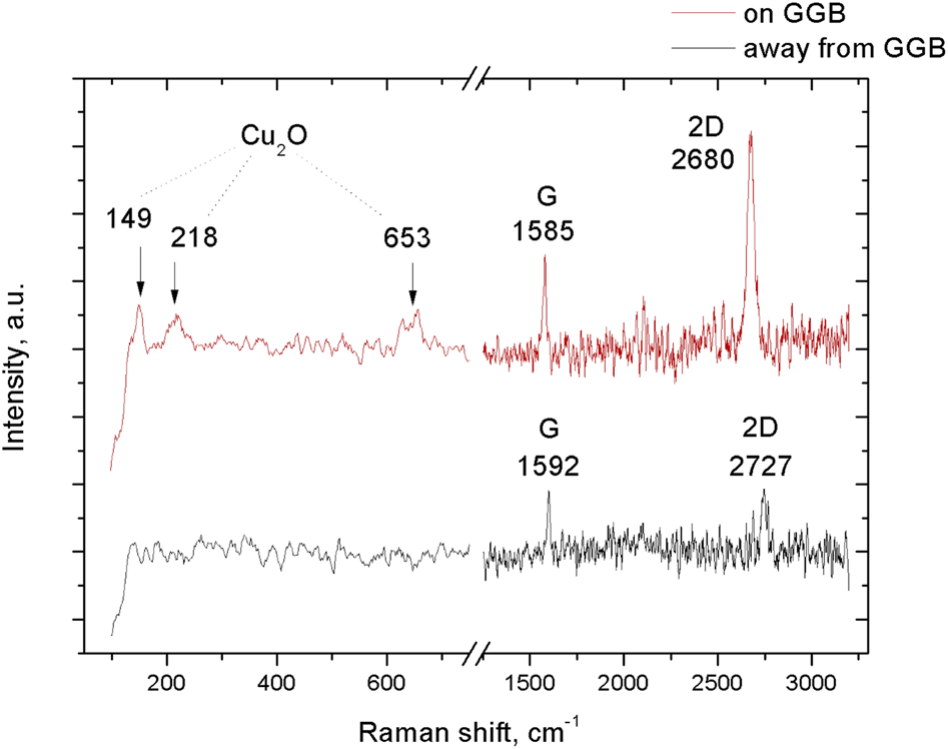
Raman spectra taken with an excitation wavelength of 514 nm on a graphene-on-copper sample, after it had been exposed to air for 2 months. (Upper spectrum, red) Measurement on a protruding GGB region. (Lower spectrum, black) Reference measurement far away from the GGBs. Note the extra peaks in the upper spectrum, at 149, 218, and 653 cm−1, associated with Cu_2_O. Also note the ‘red shifts’ of the G- and 2D-peaks on the oxidized substrate and the absence of a D-peak at 1350 cm^−1^.

In order to acquire direct evidence for the local oxidation of the copper substrate, we measured Raman spectra on the modified areas, close to a GGB, and compared these with spectra taken on the unmodified areas, at distances from the nearest GGBs of at least 20 μm. An example of a Raman spectrum on a protruding region that decorated a GGB, is presented as the upper graph in Fig. 3. This spectrum should be compared with the lower graph, taken on the same sample, on a region far away from GGBs that seems unaffected by the two months of air exposure. The three peaks in the upper spectrum at 149, 218 and 653 cm^−1^ are the signature of Cu_2_O,^29^ and support our interpretation that the copper substrate is oxidized at the location of the protrusion. These peaks are absent in the lower spectrum, in accordance with the idea that the graphene protected most of the copper substrate from oxidation. We find that on the oxidized copper, the G- and 2D-peaks of the graphene are both present, indicating that the graphene is still there and is not oxidized. The G- and 2D-peaks are both ‘red-shifted’ with respect to the corresponding peaks for ‘regular’ graphene on unmodified copper (Fig. 1 and lower graph in Fig. 3), from 1592 to 1585 cm^−1^ for the G-peak, and from 2727 to 2680 cm^−1^ for the 2D-peak. Previously, a similar red-shift of the characteristic Raman peaks was reported for graphene-coated copper samples that were oxidized on purpose with ultraviolet radiation (UV) in an oxygen atmosphere.^28,30^ Interestingly, a red-shift of the characteristic peaks is known to originate from tensile strain in the graphene. In our case, there is a natural reason for such strain in view of the increased volume below the graphene due to the oxidation of the copper.^30^ Using the observations of ref. 31 as our frame of reference, we associate the red-shifts in Fig. 3 to a tensile change in the strain of the graphene between 0.5 and 1.5%. There is also a change in the intensity ratio of the 2D- and G-peaks, which equals *I*_2D_/*I*_G_ = 2.3 on the oxidized copper (upper graph in Fig. 3), which is much higher than the ratio of approximately unity for unoxidized areas (*cf.*Fig. 1 and lower graph in Fig. 3).

In spite of the change in strain, the Raman spectrum on the oxidized region did not contain a detectable D-peak, showing that the substrate oxidation and the internal strain in the graphene overlayer were not accompanied by structural defects in the graphene.

**Fig. 4:**
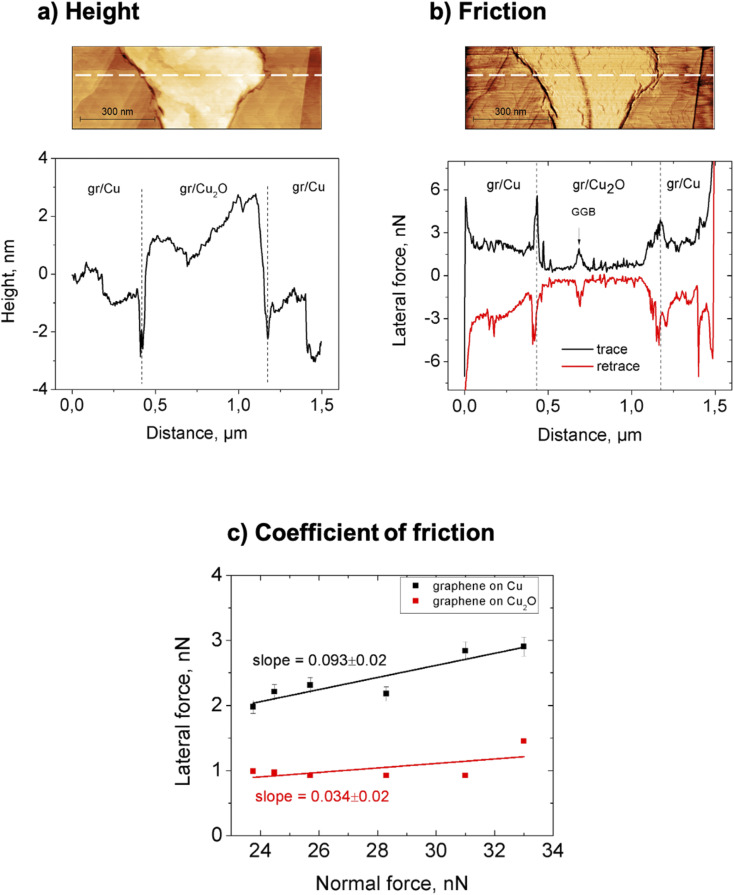
Quantitative comparison of friction properties of graphene on copper and graphene on Cu_2_O. (a) Regular AFM height image, zoomed in on a 1.5 × 0.5 μm^2^ region of graphene-covered copper sample with an oxidized region in the center, combined with a single height profile taken along the dashed line in the image. (b) Lateral force image of the same area, taken in the retrace (right to left) direction at a normal force of F_N_ = 23.7 nN, combined with two lateral force profiles, taken in the trace and retrace directions along the dashed line in the image (same location as the dashed line in (a)). (c) Dependence of the (absolute value of the) lateral force on the normal force for graphene on copper and for graphene on Cu_2_O, measured along the same dashed line in (a) and (b); the slopes indicate the coefficient of friction.

Using AFM, we measured the local topography and the frictional behavior of the graphene-on-copper samples, both before and after prolonged air exposure. Fig. 4a and b present a combination of height and lateral-force measurements on a 1.5 × 0.5 μm^2^ area of the exposed sample, centered around an oxide region with a GGB in the middle; the GGB runs approximately vertically in the middle of the image and stands out most clearly in the lateral force map of panel (c). The AFM measurements were conducted in ambient at a normal force *F*_N_ in the range from 23.7 to 33.0 nN, using a cantilever with a normal spring coefficient of 0.09 N m^−1^ and a lateral spring coefficient of 38 N m^−1^. The graphs in Fig. 4a and b show typical height and lateral force curves; they were taken along the white dashed lines in the two corresponding images. The height measurements show that the oxide region is higher than the surrounding, unoxidized surface by 2 to 4 nm. Interestingly, the measurements of the lateral force *F*_L_ in panel (b) indicate that the friction force experienced by the AFM tip on the graphene on oxidized copper is significantly lower than that on the unoxidized copper. This can be recognized from the contrast in the friction force image, the brighter color corresponding to a lower absolute value of the lateral force, and from the changes in both the trace and retrace curves in corresponding lateral force graph, the absolute value for the lateral force for both curves being minimal over the oxidized region. Note, that a local maximum is observed in the lateral force across the GGB (Fig. 4b).

Additional evidence for differences in frictional behavior between the oxidized and unoxidized regions comes from measurements of the lateral force as a function of the normal force, shown in Fig. 4c. For both the oxidized and unoxidized regions, the lateral forces, plotted in Fig. 4c, were calculated as the average lateral force in measurements such as those in Fig. 4b, over regions that were neither near the transitions between copper and oxide nor near other, significant topographical features that could lead to artifacts in the measured lateral force. The error margins in Fig. 4c indicate the standard deviations on the measured averages, the error margins on the data for the oxidized regions (red squares) falling just within the symbol size.

From Fig. 4c, we recognize that not only the friction force is lower on the oxidized regions, but also the friction coefficient, *i.e.*, the slope *μ* = *dF*_L_/*dF*_N_. This can be seen directly from the two linear fits in Fig. 4c. We find *μ*_ox_ = 0.034 ± 0.02 for the friction on graphene on oxidized copper and *μ*_unox_ = 0.093 ± 0.02 for the friction on graphene on bare copper.

### 2.2. Puckering as a qualitative interpretation

It was suggested before that the lateral displacement of the tip that slides over graphene can make graphene locally lift off from the substrate, forming a bulge in front of the tip, when the tip interaction with the graphene is sufficiently strong with respect to the graphene-substrate adhesion. Systematic AFM studies for different layered materials showed that this puckering is a universal phenomenon for weakly adhering or freely suspended materials.^3,8,15,32,33^ In turn, it is suppressed for strongly adhering substrates.^15^

We interpret our experimental finding that friction is low on oxidized copper as the consequence of the reduction of the puckering effect on Cu_2_O with respect to unoxidized copper. Graphene has been shown to exhibit strong adhesion on other oxides, Al_2_O_3_ and Si_2_O, nearly equally strong as that on Fe.^21^ As the adhesion is known to grow with increasing dielectric constant of the substrate and the value of *ε*_Cu_2_O_ = 18.1 for Cu_2_O is much higher than that for Si_2_O of *ε*_Si_2_O_ = 3.5,^34^ we could expect the adhesion of graphene to be even stronger on Cu_2_O than on Si_2_O.

In the next section we use DFT calculations to explore whether or not the adhesion between graphene and Cu_2_O differs enough from that between graphene and bare copper to support the idea that puckering is suppressed by the substrate oxidation. And secondly, we aim at explaining why this suppression of puckering is accompanied by such a consistent reduction of friction.

In addition to the increase in graphene-substrate adhesion, we can imagine two further contributions to reduced puckering of graphene on the oxide. The first is that a higher substrate roughness may reduce adhesion between the tip and the graphene and thereby suppress the puckering effect between them.^35^ We measured an rms roughness for the graphene on Cu_2_O of 0.7 nm, indeed higher than the roughness of 0.4 nm for graphene on bare copper. The graphene layer on bare copper presents higher puckering than on oxidized copper, even if the latter substrate is slightly rougher. This counter-intuitive effect is explained by the different level of adhesion estimated by DFT calculations, which makes it much easier for graphene to deform out-of-plane deformation much easier on the weakly adherent substrate. The second is that the volume increase due to local oxidation of the substrate introduces tensile strain in the graphene overlayer, stretching out wrinkles and making the graphene less prone to wrapping around the tip apex. Neither of these extra contributions is expected to be sufficient to explain the dramatic decrease in friction.

### 2.3. DFT calculations

**Fig. 5:**
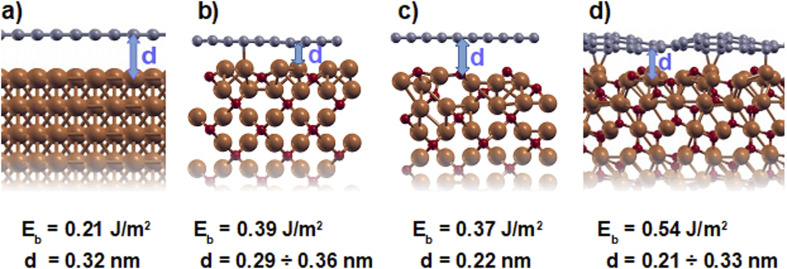
DFT results for the geometries, binding energies, *E*_b_ and bonding distances, *d* for graphene on (a) Cu(111), (b) Cu_2_O(100) : Cu, (c) Cu_2_O(100) : O and (d) Cu_2_O(111).

The optimized structures of graphene adsorbed on the four considered substrates are shown in Fig. 5, along with the calculated binding energies, *E*_b_ and distances, *d*. The result for graphene on bare copper, which falls in the typical range of physisorption interactions, is in agreement with previous theoretical calculations.^36–38^ We are not aware of any previous theoretical calculations of graphene adsorption on copper oxide. Our calculations reveal that oxidation of the copper leads to an increase of the adhesion of graphene to the substrate. This result, which is independent of the surface orientation of the oxide, can be explained by considering the different reactivity of the Cu atoms in the clean and oxidized samples: the Cu atoms of the elemental substrate have fully occupied d orbitals and are less reactive when exposed at the surface than the Cu atoms at surfaces of the three oxide substrates, where the re-hybridization, caused by the interaction with the oxygen, results in a number of under-coordinated sites at the surface that are more reactive.^39^ In the Cu_2_O(111) substrate, in particular, the under-coordinated copper atoms tend to form chemical bonds with the carbon atoms above, which introduces minor out-of-plane deformations in the graphene layer and increases its binding energy by more than a factor two with respect to pure copper.

The results reported in Fig. 5 were calculated within the LDA approximation. Those obtained using other exchange functionals, also including the van der Waals interactions, are reported in the ESI.† Importantly, the trend observed for graphene binding on the different substrates is common to all considered functionals.

The difference in binding energies that our calculations revealed for graphene on elemental and oxidized Cu, can be related to the difference in frictional properties observed in our experiments. In particular, we should expect that the increase in binding energy occurring upon oxidation of the Cu substrate suppresses the puckering effect. Indeed, as was suggested to explain the different frictional behavior of multilayer graphene films on different substrates,^15^ a reduction of graphene puckering results in a friction decrease. Here, we will inspect the relation between puckering and friction in further detail. As mentioned in the introduction, two main hypotheses were proposed in this context, namely the increase of contact area caused by the formation of a bulge in front of the tip and the increase of commensurability between the tip and graphene, enabled by the flexibility of graphene on weakly attracting substrates. It seems unrealistic that either of these effects or even a combination of the two could lead to the observed reduction in friction by nearly a factor three.

Here we introduce a new explanation, based on the analysis of the chemical reactivity of graphene as a function of its curvature. In particular, we propose that graphene puckering is accompanied by a change in the hybridization of the carbon atoms from sp^2^ to sp^3^-like, with a corresponding increase of reactivity due to the appearance of dangling bonds. This hypothesis is in agreement with the observation that water molecules dissociatively chemisorb on curved graphene regions, while they weakly physisorb on flat graphene, as revealed by QM/MM dynamic simulations performed by our group.^40^

**Fig. 6:**
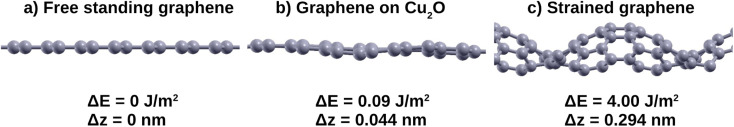
DFT results for the geometries, surface energies and out-of-plane deformations of graphene with three different curvatures. (a) Flat, free-standing graphene, (b) mildly curved graphene on Cu_2_O(111) and (c) free-standing graphene with a strong curvature, imposed by a 19% lateral compression. The surface energies are expressed as the difference with respect to the value for the free-standing graphene layer.

To verify this hypothesis, we calculate the effect of various curvatures on the surface energy of free-standing graphene. As shown in Fig. 6b, where the considered curvature is the same as that of Fig. 5d, a small out-of-plane deformation, induced by the interaction with the Cu_2_O(111) substrate, does not result in a significant increase of surface energy with respect to flat graphene (Fig. 6a). By contrast, the enforced 0.3 nm puckering in Fig. 6c is accompanied by a huge increase of surface energy by 4 J m^−2^. The curved graphene structure of Fig. 6c was obtained by structural optimization of a free-standing graphene system composed of 50 carbon atoms in a supercell of which the in-plane area was deliberately made 19% smaller than the equilibrium size. Even though this highly strained situation should be regarded as an exaggeration with respect to the typical configurations that should be expected in the case of the tip-contact-induced puckering, the example of Fig. 6c is useful to emphasize the enormous increase in energy that is introduced in the graphene by the puckering distortion.

**Fig. 7:**
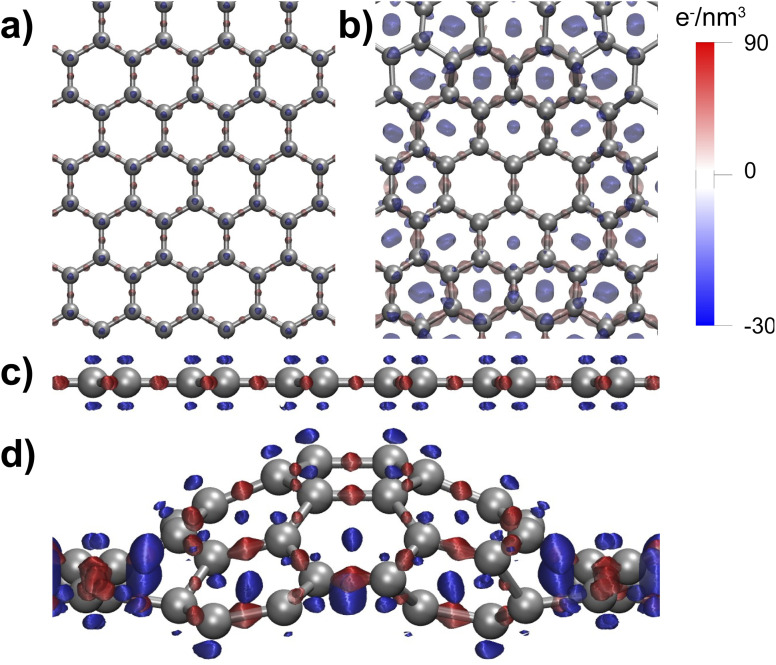
DFT results for the electronic charge redistribution, when the interactions are ‘switched’ on between the carbon atoms in flat graphene (panels a and c) and in puckered graphene (panels b and d). Red and blue colors indicate accumulation and depletion of electron density, respectively. Whereas the redistribution for the flat layer is illustrative for the sp^2^ bonding in the graphene network, the depletion of charge in the centers of the hexagonal rings is indicative of the loss of the p orbitals and the local transition from an sp^2^ to an sp^3^ configuration.

**Fig. 8:**
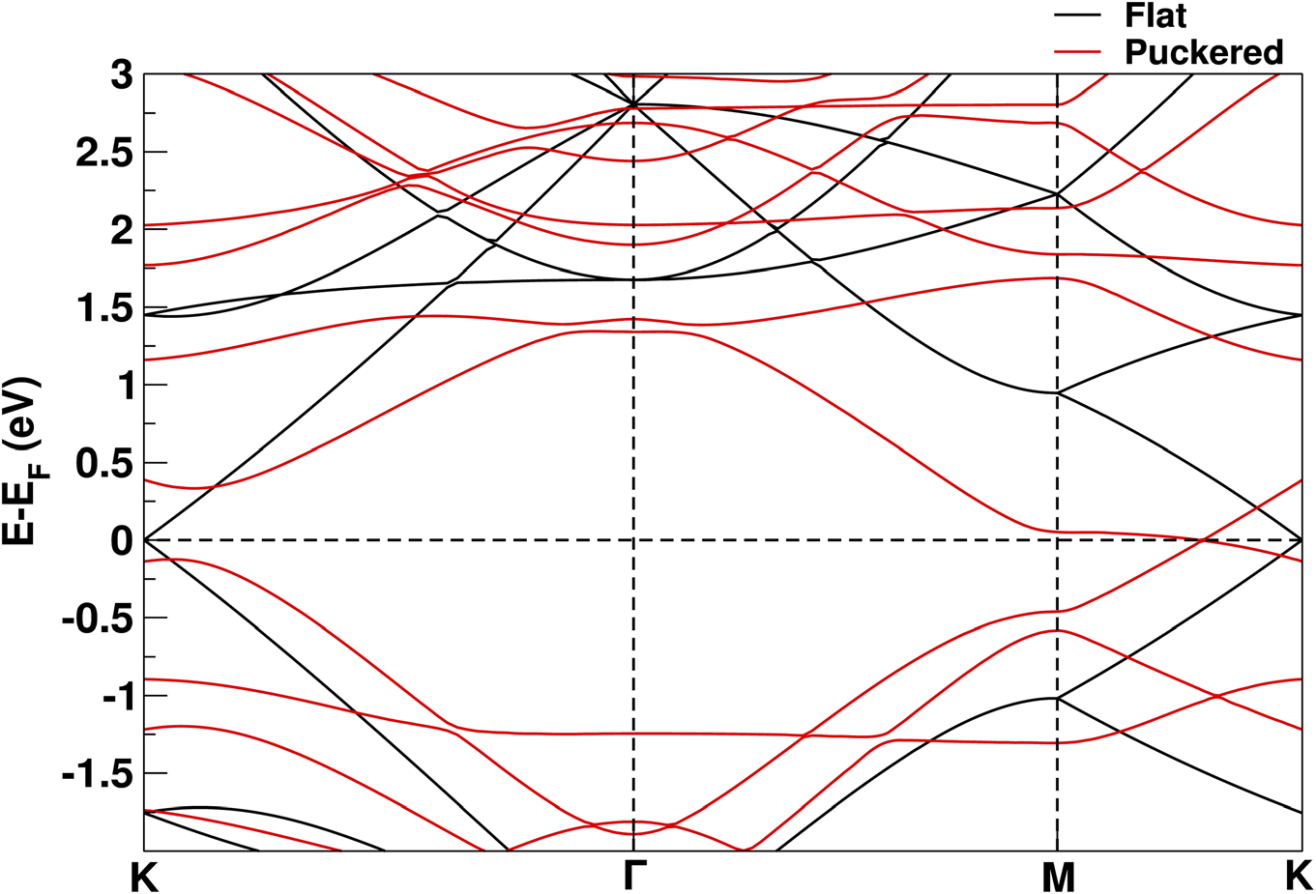
Electronic band structure for flat (black) and puckered (red) graphene, along three high-symmetry directions in the first Brillouin zone of the graphene. In addition to distortion-induced changes in the precise eigenenergies, the graph shows that the puckering of the graphene leads to a heavy distortion of the characteristic Dirac cones around the *k*-point and the accompanying opening of a significant energy gap instead of the Dirac points of flat graphene. These changes in band structure are reflected in the changes in charge density distribution of Fig. 7 and the enhanced reactivity of the curved graphene.

The increase in energy and, hence, in reactivity of the graphene stems from a redistribution of the electronic charge within the carbon network. To visualize this effect, we calculated the charge rearrangements occurring for flat and puckered graphene layers, when they are formed from a hypothetical arrangement of initially non-interacting C atoms, located in the same positions. As shown in Fig. 7, the carbon–carbon interaction causes a charge accumulation (in red) along the graphene bonds and a corresponding depletion (in blue) from other regions between the atoms, *e.g.*, from the center of the graphene rings, where the electron density distributions of the initially non-interacting atoms overlap. By comparing the top and side views in Fig. 7b and d for the puckered structure with the corresponding views in Fig. 7a and c for the flat arrangement, we recognize that the bonds along the slopes of the curved graphene contain a larger amount of charge than those in flat graphene and that a larger charge depletion occurs above and below these atoms, indicating that the p orbitals have disappeared and the hybridization can no longer be regarded as sp^2^. This change in electronic densities is also reflected in Fig. 8 in the band structure of the curved graphene, in which a gap is opened at the *k*-point that distorts the Dirac cones and removes the Dirac point, the two defining features in the band structure of flat graphene.

The higher reactivity of curved graphene is not a new concept in the literature: this system has already been proposed as an efficient configuration for hydrogen storage.^41,42^ As further evidence of its increased reactivity, we show in the ESI† that the adsorption energy of hydrogen molecules on flat graphene (+0.91 eV per H_2_ molecule) is quite different from that on curved graphene (−0.36 eV per H_2_ molecule) and certainly more in favor of the latter. Therefore, our calculations and experimental data suggest a new hypothesis regarding the puckering effect present in nanotribology experiments. Instead of increasing the contact area between the tip and the substrate, we propose that it is the increased charge transfer of curved graphene that leads to an increased reactivity that can explain the larger friction between the tip and the sample observed in the experiments.

## 3. Conclusions

Based on our combined experimental and theoretical study, we conclude that the local oxidation of the Cu substrate underneath a monolayer of graphene leads to a significant increase in the adhesion of the graphene to the substrate and an according reduction in the puckering effect. The accompanying decrease in measured friction force is so large that it cannot be ascribed solely to changes in contact area or local commensurability. As the dominant contribution to the large difference in friction we identify the change in electronic structure that the curves locally introduce in the puckered graphene and the corresponding changes in the reactivity and, thus, the adhesion and friction between the graphene and the tip of the friction force microscope. Since the geometry in these single-asperity experiments may be similar to the local configurations in extended, dry contacts that are lubricated by a single monolayer of graphene, we should expect a similarly strong dependence of the lubricating qualities of such contacts on the oxidation state of the substrate on which the graphene resides. This should be of relevance in the context of advanced MEMS and NEMS devices where graphene lubrication is applied or considered.

## 4. Materials and methods

### 4.1. Experimental

For our experiments, we used high-quality, single-monolayer graphene samples obtained from Applied Nanolayers B.V. (ANL).^43^ The graphene was grown by chemical vapor deposition (CVD) on thin polycrystalline copper films. These copper films were formed by sputter deposition of copper on sapphire (111) wafers (diameter 51 mm, thickness 750 μm). The thickness of the copper film was 1 μm. The copper grains in the film showed a strong preference for the (111) surface orientation. The graphene-on-copper samples were inspected at various stages in the experiment with optical microscopy and with Renishaw Raman spectroscopy with an excitation wavelength of 514 nm. These Raman spectroscopy measurements were conducted with a spot size of approximately 1 μm that could be located with respect to the features in the optical microscopy images with a precision of several micrometers. More precise topography measurements and lateral force measurements were done with a Bruker Icon AFM, using V-shaped Si3N4 probes (DNP-10). The normal and lateral force calibration was carried out according to the method discussed in ref. 44 and 45.

### 4.2. Theoretical

We performed density functional theory (DFT) calculations within the plane-waves/pseudopotential scheme,^46^ considering different approximations for the exchange-correlation functional, namely the local density approximation (LDA), the generalized gradient approximation due to Perdew, Burke and Ernzerhof (PBE),^47^ and the PBE-D scheme. The latter includes van-der-Waals interactions within the semi-empirical approach proposed by Grimme.^48^

We studied graphene adsorption both on copper and on copper oxide. In the case of copper, we considered the (111) surface, as the most stable surface of this material and the preferred one, as found in the experiments, while for Cu_2_O we considered three different surface orientations, in order to analyze the effects of different surface stoichiometries. In particular, we considered the Cu_2_O(100) : Cu surface, terminating with a copper layer; the Cu_2_O(100) : O surface, terminating with an oxygen layer, and the Cu_2_O(111) surface, terminating with a layer that contains both copper and oxygen in the stoichiometric ratio 2 : 1. The relative stabilities of these surfaces change as a function of the chemical potential of oxygen.^39^

Graphene adsorption on these copper and copper oxide substrates was studied using periodic supercells containing a substrate slab and a vacuum region of 1 nm and 1.5 nm thickness, respectively. The in-plane size of the supercells was chosen according to the lattice mismatch between the graphene layer and the substrate: the (1 × 1) cell was used for Cu(111), a (3 × 3) cell for Cu_2_O(100), and a (2 × 2) cell for the Cu_2_O(111) surface. For all considered systems, the residual lattice mismatch between graphene and the substrates was around 2.5%. We calculate the graphene binding energy on a substrate as *E*_b_ = (E_substrate_ + *E*_grap_ − *E*_tot_)/*A*, where *A* is the in-plane area of the considered supercell, *E*_substrate_ (*E*_grap_) is the total energy of a supercell containing the isolated substrate (graphene) and *E*_tot_ is the total energy of the same supercell containing the adsorbed graphene on the considered substrate. Further computational details are reported in the ESI,† along with the results concerning bulk structures.

## Author contributions

P. V. Antonov: investigation, conducting experimental work, writing-original draft. P. Restuccia: investigation, performing theoretical calculations, writing-review & editing. M. C. Righi: conceptualization of the theoretical work, supervision, writing-review & editing. J. W. M. Frenken: conceptualization of the experimental work, supervision, writing-review & editing.

## Conflicts of interest

The authors declare that they have no known competing financial interests or personal relationships that could have appeared to influence the work reported in this paper.

## Supplementary Material

NA-004-D2NA00283C-s001
