# Supplementary material for: Attractive curves: the role of deformations in adhesion and friction on graphene
Source: Nanoscale Adv. 2022 Sep 14;4(19):4175–84. doi: 10.1039/d2na00283c (PMC9514564; doi:10.1039/d2na00283c)
Supplement: NA-004-D2NA00283C-s001 [file NA-004-D2NA00283C-s001.pdf]

Electronic Supplementary Material (ESI) for Nanoscale Advances.
This journal is © The Royal Society of Chemistry 2022

Supporting Information Attractive curves: the role of deformations in adhesion and friction on graphene

P.V. Antonov[1,#], P. Restuccia[2], M.C. Righi[2*] and J.W.M. Frenken[1,3*]
[1]Advanced Research Center for Nanolithography, Science Park 106, 1098 XG Amsterdam, The Netherlands
[2]Department of Physics and Astronomy, University of Bologna, Viale Berti Pichat 6/2, 40127, Bologna, Italy
[3]Institute of Physics, University of Amsterdam, Science Park 904, 1098 XH Amsterdam, The Netherlands
[#]Present address: ASML Netherlands B.V., PO Box 324, 5500 AH Veldhoven, The Netherlands
[*]Corresponding authors: frenken@arcnl.nl and clelia.righi@unibo.it

Cu_2O bulk calculations

Before calculating the binding energy of graphene on copper oxide, we performed preliminary test calculations in order to evaluate the bulk properties of Cu_2O within the exchange-correlation functionals selected in this work. The bulk structure of Cu_2O consists of a cubic unit cell composed of 4 copper atoms and 2 oxygen atoms that are located on two different sublattices: the Cu atoms reside on a face-centered cubic sublattice (Fig. S1a), while the O atoms are located on a body-centered cubic sublattice (Fig. S1b). The experimental lattice parameter is equal to 0.427 nm, while the bulk modulus is equal to 110 GPa. The results of calculations with LDA and PBE are both in good agreement with the experimental data: the lattice parameter and the bulk modulus within the LDA scheme are equal to 0.418 nm and 137 GPa, respectively, while these quantities for the PBE scheme are equal to 0.431 nm and 107 GPa, respectively. For both functionals we found an optimal energy (charge density) cutoff of 408.17 eV (3265.37 eV). The optimal sampling of the Brillouin zone of the bulk structure was realized by a 9 x 9 x 9 Monkhorst-Pack grid. This optimal k-point density was used for all the following calculations.

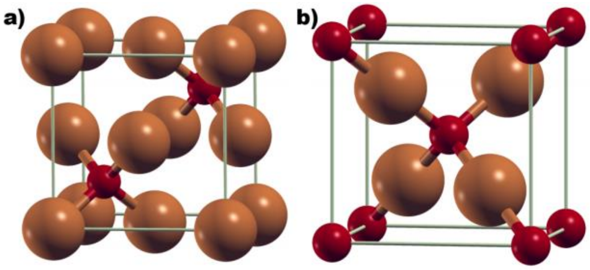

Figure S1. *Two different views of the same Cu_2O bulk structure. The cubic unit cell is chosen to have Cu atoms at its corners in panel a), and to have O atoms at its corners in panel b).*

Geometries used for graphene on the different substrates

As reported in the main manuscript, we calculated the binding energy of graphene on different substrates. For graphene on Cu(111) we chose a 1 x 1 supercell, containing a single graphene layer adsorbed on a six-layer thick slab of copper, with a total of 8 atoms. The graphene/Cu_2O(111) system was modeled

with a 2 x 2 supercell, containing two graphene layers adsorbed on a five-trilayer thick slab of $Cu_2O(111)$, with a total of 220 atoms. For the system composed of graphene adsorbed on $Cu_2O(100)$, we used two graphene layers adsorbed on a 3 x 3 supercell of $Cu_2O(100)$ that contained a 15-atom-layer slab: the system with the $Cu_2O(100)$ substrate terminated with Cu atoms had a total of 327 atoms, while the one with substrate terminated with oxygen contained 318 atoms.

Results of binding energy and average distance of graphene on copper and copper oxide for different exchange-correlation functionals

The following tables show the results for the binding energy (Table S1) and the average distance between the graphene layer and the substrate (Table S2) for the different exchange-correlation functionals used in this study. These results show that the PBE-D scheme produces results with similar behavior for the variations of the binding energy and the average distance compared to LDA, with the binding energy between graphene and copper oxide being higher than that between graphene and bare copper, albeit that the difference is less pronounced within the PBE-D calculations than for the LDA calculations. These results confirm that graphene attaches better on the oxidized surface, regardless of the selected functional used in the simulation. We found that the simple PBE approximation fails to properly reproduce the binding energy of graphene on both substrates, yielding values that are more than one order of magnitude lower than those obtained within the LDA and PBE-D schemes. This is a well-known limitation of the PBE approximation, which is due to the absence of dispersion forces in this scheme.

Table S1. *Binding energy (J/m2) of graphene on different substrates within the considered exchange/correlation functionals.*

System	LDA	PBE	PBE-D
**Gr/Cu(111)**	0.21	0.009	0.55
**Gr/Cu_2O(111)**	0.54	0.008	0.63
**Gr/Cu_2O(100):Cu**	0.39	–	–
**Gr/Cu_2O(100):O**	0.37	–	–

Table S2. *Average adsorption distance (nm) of graphene on different substrates within the considered exchange/correlation functionals.*

System	LDA	PBE	PBE-D
**Gr/Cu(111)**	0.32	0.42	0.30
**Gr/Cu_2O(111)**	0.21 ÷ 0.33	0.35 ÷ 0.42	0.25 ÷ 0.31
**Gr/Cu_2O(100):Cu**	0.29 ÷ 0.36	–	–
**Gr/Cu_2O(100):O**	0.22	–	–

Results of hydrogen adsorption over flat and curved graphene

To evaluate the reactivity of flat and curved graphene, we performed additional DFT calculations for the adsorption of hydrogen atoms from the gas phase onto either a flat or a curved, free-standing graphene layer. In both cases, the unit cell was composed of 50 carbon atoms in the graphene layer, on which six

hydrogen atoms (three H_2 molecules) were adsorbed. For these calculations, we employed the same simulation cell as in panels a) and c) of Fig. 6, with the bent graphene layer again obtained by reducing the in-plane size area by 19% compared to the flat one. The adsorption energy was computed as $E_b = (E_{graphene} + n_{mol} \cdot E_{H_2} - E_{tot})/n_{mol}$, where n_{mol} is the number of H_2 molecules present in the simulation cell, $E_{graphene}$ (E_{H_2}) is the total energy of a supercell containing the isolated graphene (H_2 molecule) and E_{tot} is the total energy of the same supercell containing the adsorbed H atoms on the considered graphene layer. The results in Fig. S2 show the significant difference in the adsorption energy between the two considered systems: while hydrogen adsorption is energetically unfavorable on flat graphene, the energy actually rising by +0.91 eV per H_2 molecule, the curved graphene has strong adsorption energy of -0.36 eV per H_2 molecule. This indicates a significant change from the flat graphene that is completely unreactive towards hydrogen adsorption to a high reactivity of curved graphene.

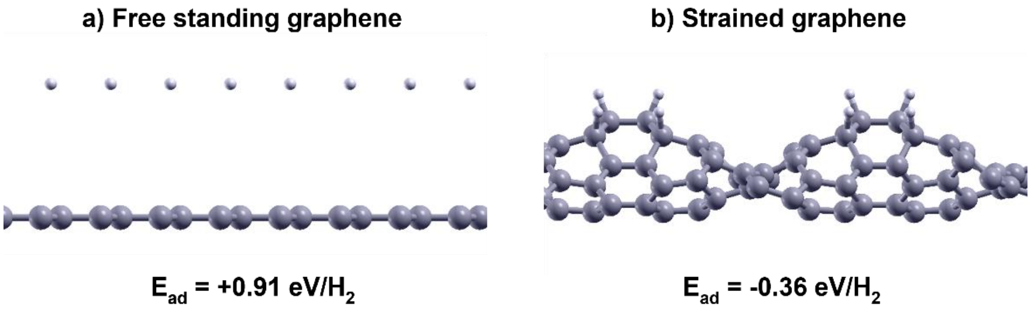

Figure S2. *Ball and stick representation of hydrogen adsorption on flat and curved graphene. Gray and white balls represent carbon and hydrogen atoms, respectively. Panel a) shows that the dissociative adsorption of hydrogen molecules on flat, free-standing graphene requires an increase in total energy and results in a large distance between the hydrogen atoms and the graphene layer. In panel b) we see the dramatic effect of curvature on the reactivity of graphene. The bending leads to intimate bonding of the hydrogen to the graphene, as is illustrated by the short bonding distance and the strongly negative energy for dissociative adsorption.*
